# Associations between Passive Maternal Smoking during Pregnancy and Preterm Birth: Evidence from a Meta-Analysis of Observational Studies

**DOI:** 10.1371/journal.pone.0147848

**Published:** 2016-01-25

**Authors:** Hong Cui, Ting-Ting Gong, Cai-Xia Liu, Qi-Jun Wu

**Affiliations:** 1 Department of Obstetrics and Gynecology, Shengjing Hospital of China Medical University, Shenyang, China; 2 Department of Clinical Epidemiology, Shengjing Hospital of China Medical University, Shenyang, China; National Taiwan University, TAIWAN

## Abstract

Previous studies investigating the relationship between passive maternal smoking and preterm birth reveal inconsistent results. We conducted the current meta-analysis of observational studies to evaluate the relationship between passive maternal smoking and preterm birth. We identified relevant studies by searching PubMed, EMBASE, and ISI Web of Science databases. We used random-effects models to estimate summary odds ratios (SORs) and 95% confidence intervals (CIs) for aforementioned association. For the analysis, we included 24 studies that involved a total of 5607 women who experienced preterm birth. Overall, the SORs of preterm birth for women who were ever exposed to passive smoking *versus* women who had never been exposed to passive smoking at any place and at home were 1.20 (95%CI = 1.07–1.34,*I*^2^ = 36.1%) and 1.16 (95%CI = 1.04–1.30,*I*^2^ = 4.4%), respectively. When we conducted a stratified analysis according to study design, the risk estimate was slightly weaker in cohort studies (SOR = 1.10, 95%CI = 1.00–1.21,n = 16) than in cross-sectional studies (SOR = 1.47, 95%CI = 1.23–1.74,n = 5). Additionally, the associations between passive maternal smoking and preterm birth were statistically significant for studies conducted in Asia (SOR = 1.26, 95%CI = 1.05–1.52), for studies including more than 100 cases of preterm birth (SOR = 1.22, 95%CI = 1.05–1.41), and for studies adjusted for maternal age (SOR = 1.27,95%CI = 1.09–1.47), socioeconomic status and/or education (SOR = 1.28, 95%CI = 1.10–1.49), body mass index (SOR = 1.33, 95%CI = 1.04–1.71), and parity (SOR = 1.27, 95%CI = 1.13–1.43). Our findings demonstrate that passive maternal smoking is associated with an increased risk of preterm birth. Future prospective cohort studies are warranted to provide more detailed results stratified by passive maternal smoking during different trimesters of pregnancy and by different types and causes of preterm birth.

## Introduction

Preterm birth, which is birth before 37 completed weeks of gestation, is a leading cause of neonatal death worldwide. Approximately 15% of preterm infants die within one month after birth [1]. In most countries, the rates of preterm birth have been increasing in recent decades and this represents a primary obstacle to the World Health Organization’s Millennium Development Goal 4, which is to reduce childhood mortality [2–3]. Therefore, it is important to identify modifiable risk factors that may lead to the primary prevention of preterm birth.

Active smoking is well-established as a contributing factor to preterm birth. There is increasing scientific and regulatory concern for the role that passive smoking, which is the exposure to environmental tobacco smoke or second-hand smoke, may play in preterm birth, possibly due to the same biological mechanisms as active smoking [4–5]. However, evidence from observational studies is conflicting [6–8]. Some studies provided evidence that passive maternal smoking increased the risk of preterm birth, but others found no association. A recent meta-analysis, which summarized the results of studies published prior to May 2009, found no effect of passive maternal smoking on preterm birth (pooled risk estimate = 1.07, 95% confidence interval (CI): 0.93–1.22) [8]. However, this meta-analysis focused not only on preterm birth but also on other perinatal outcomes including birth weight, infant length, and congenital anomalies. Therefore, the authors only reported summarized risk estimates of these outcomes instead of conducting subgroup analyses to find the source of heterogeneity. Additionally, it is not clear whether the findings of the study were robust in the subgroup and sensitivity analyses. Several additional epidemiological studies of preterm birth and passive maternal smoking have been published during the past 5 years [9–14]. For example, Qiu et al [9] conducted an analysis in a birth cohort study including 10,095 non-smoking women who delivered a singleton live birth in China; the findings supported a positive aforementioned association, especially very preterm birth, which is birth between 28 and 31 weeks of gestation, regardless of whether the preterm birth was medically indicated or spontaneous. Khader et al [12] conducted a cross-sectional study of 8,490 women and demonstrated that exposure to passive smoking during pregnancy was significantly associated with an increased odds of preterm delivery. However, Andriani et al [14] conducted the first national prospective longitudinal cohort study of passive maternal smoking and preterm birth in Indonesia and found no significant associations.

A large portion of women in the general population are exposed to passive smoking [15], so even a small association between passive smoking and preterm birth may pose a substantial public health burden [6]. We conducted a meta-analysis to obtain overall summary estimates for associations between passive maternal smoking and preterm birth and to evaluate heterogeneity among the results.

## Materials and Methods

### Literature search

We performed a comprehensive search of articles published through February 28, 2015 by searching PubMed, EMBASE, and Web of Science databases. The following terms were used in the electronic search: (passive smoking, environmental tobacco smoke, second hand, cigarette) and (preterm birth, prematurity). We also manually searched the references cited in the retrieved articles. This meta-analysis was planned, conducted, and reported in adherence with the Preferred Reporting Items for Systematic Reviews and Meta-Analyses (PRISMA) guidelines [16].

### Eligibility criteria

Studies were selected and excluded by 2 independent investigators (Q-JW and T-TG). Published articles were included according to these selection criteria: 1) the study used an observational study design (e.g., cohort study, case-cohort, nested case-control, case-control, or cross-sectional study); 2) the study provided information on passive maternal smoking (exposure at home, work, or another place) as the exposure; 3) the study reported preterm birth (defined as delivery before 37 completed weeks or 259 days of gestation from first day of the last menstrual period) as the outcome; and 4) the study reported usable risk estimates between passive maternal smoking and preterm birth.

Published articles were excluded according to the following criteria: 1) the study was a review without original data, an ecological study, an editorial, or a case report; 2) the study reported the risk estimates for the highest category relative to the lowest category of passive maternal smoking instead of exposure to passive maternal smoking; and 3) the study investigated passive maternal smoking in a certain trimester of pregnancy instead of the entire pregnancy.

### Data extraction and quality assessment

Two independent reviewers (Q-JW and T-TG) completed the data extraction using a predefined sheet. Dissimilarities were resolved by discussion between the authors. The following data were extracted from each included study: first author’s name, year of publication, country of study, study design, period of exposure measurement, number of subjects with preterm birth and sample size of the study, categories of exposure with corresponding risk estimates, and potential confounders adjusted in the primary analysis.

We used the Newcastle-Ottawa Scale (NOS) [17–21] and Agency for Healthcare Research and Quality (AHRQ) criteria [22] to assess the methodological quality of all studies included in this meta-analysis. Quality scoring might conceal important information by combining disparate study features into a single score and introduce an arbitrary subjective element into the analysis [23–25]; therefore, we evaluated the included studies on the basis of NOS and AHRQ criteria instead of scoring and categorizing the studies as “high” or “low” quality.

### Statistical analysis

Since the majority of included studies reported risk estimates as odds ratios (ORs) [9–14,26–36] and the absolute risk of preterm birth is low, we interpreted all risk estimates as ORs for simplicity. For studies [33,37] that reported risk estimates separately according to the level of passive smoking instead of reporting “yes” or “no” for any exposure, the effective-count method [38] was used to recalculate the ORs and 95% CIs. For a study [35] that reported risk estimates separately by age, we used a random-effects model to calculate an overall combined estimate before combining with the rest of the studies [39–40]. For studies [28–29,34,36,41–44] that reported the necessary data instead of providing the risk estimates directly, we used these data to calculate the crude ORs. For a study [10] that reported risk estimates separately according to exposure location, we directly combined these results with the other studies. To examine the aforementioned associations, we estimated SORs with 95% CIs by summarizing the risk estimates of each included study using fixed-effects models [45] and random-effects models [46] on the basis of heterogeneity. Heterogeneity between studies was evaluated with Cochran *Q* and *I*^2^ statistics. For the *Q* statistic, a *P*-value less than 0.1 was considered to represent statistically significant heterogeneity. For the *I*^2^ statistic, a value greater than 75% was considered to indicate significant heterogeneity; a value less than 25% indicated the absence of significant heterogeneity [45,47]. We summarized the study-specific ORs to compare women who were exposed to passive smoking during pregnancy with women who were not.

To find the possible sources of heterogeneity of the primary results, we carried out the stratified analyses according to the following study features for all studies: design of study (cohort, cross-sectional, and case-control study), study location (Asia, North America, and Europe), median number of cases (≥ 100, < 100, and unknown), time of exposure measurement (before delivery and after delivery), and potential confounders adjusted in the analyses (maternal age, body mass index, parity, and preeclampsia). Small study bias was assessed by visual inspection of a funnel plot [18,48] and by testing with Egger’s test [49] and Begg’s test [50]. All statistical analyses were conducted with Stata (version 12; StataCorp, College Station, TX).

## Results

### Literature search

**Fig 1:**
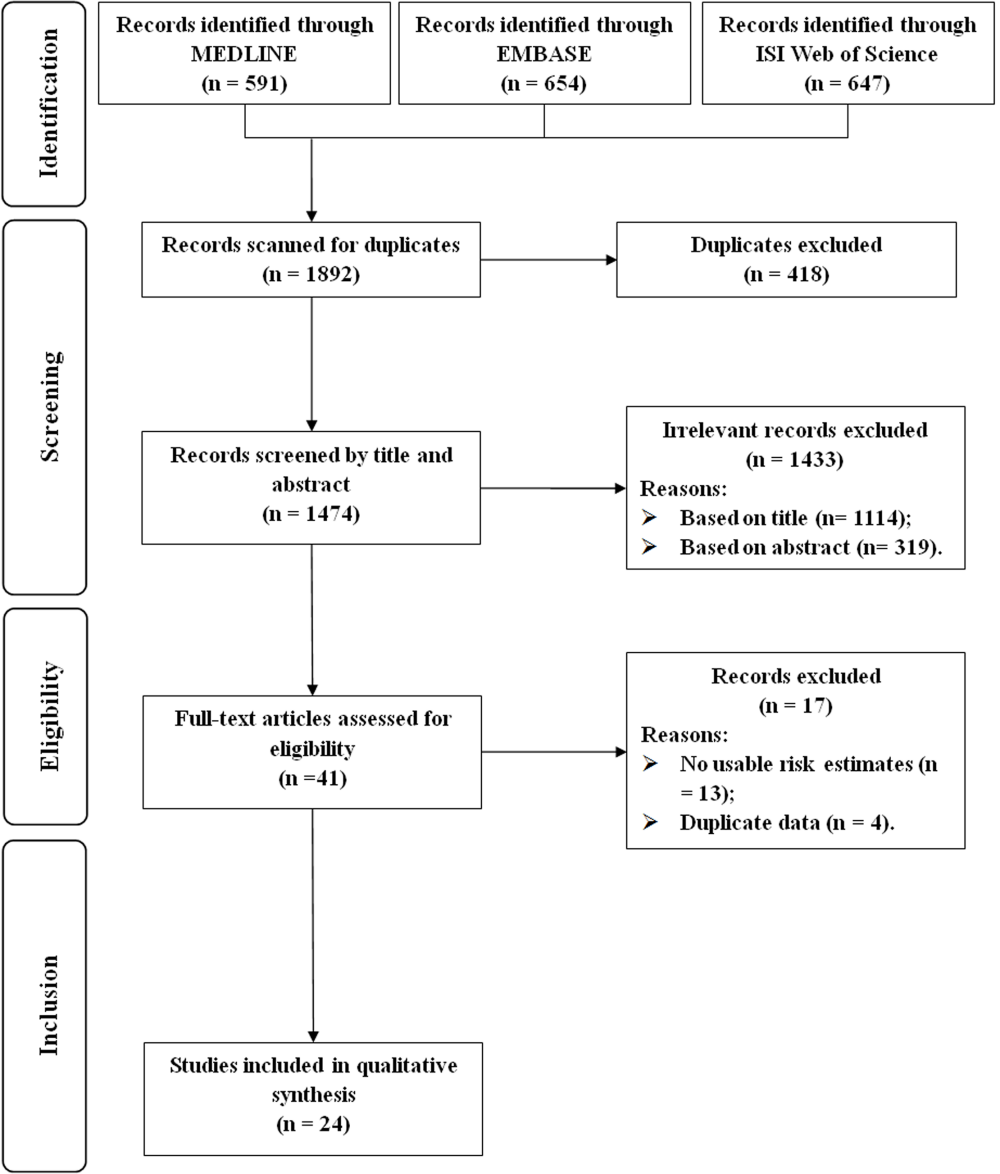
Flow-chart of study selection.

The detailed article screening processes are outlined in Fig 1. Briefly, we identified 1474 articles from the search of the 3 databases, after excluding duplicates. Of these articles, 1114 and 319 articles were excluded according to the exclusion criteria after reviewing the title and the abstract, respectively. After reviewing the full text of the remaining 41 articles, 13 and 4 articles were excluded for not reporting usable risk estimates or 95% CIs and for reporting the results of interest using the same study populations as other studies, respectively. Finally, we included a total of 24 articles that presented data on the relationship between passive maternal smoking and risk of preterm birth in this meta-analysis [9–14,26–37,41–44,51–52].

### Study characteristics and quality assessment

The characteristics of the 24 articles are described in Table 1. Together, the included studies, which were published between 1986 and 2014, represent a total of 15,764 women who experienced preterm birth. Briefly, we include 16 cohort studies [9–10,14,27–30,32,34–35,37,41–44,52], 5 cross-sectional studies [12–13,31,33,51], and 3 case-control studies [11,26,36]. Of the 24 studies, 10 were conducted in Asia [9–12,14,28–31,42], 7 were conducted in North America [13,34–37,44,51], and 7 were conducted in Europe [26–27,32–33,41,43,52]. Passive maternal smoking was measured before delivery in 11 studies [10,28–30,34,37,41–42,44,51–52] and after delivery in 13 studies [9,11–14,26–27,31–33,35–36,43]. Eleven studies [9–10,14,26–27,29,32–33,36,42,52] provided risk estimates related to passive smoking exposure at home and 4 studies [9–10,32,52] provided risk estimates of passive smoking exposure at work or another place. Most of the included studies adjusted for maternal age (n = 13), socioeconomic status and/or education (n = 12), and parity (n = 10). Few of the included studies adjusted for alcohol drinking (n = 6), body mass index (n = 6), and preeclampsia (n = 2).

**Table 1:** Characteristics of studies included in the meta-analysis.

First Author, (Reference), Year, Country	Study Design	No. of Case/Study size	Exposure categories (period of exposure measurement)	Risk Estimates(95% CI)	Adjusted factors
Andriani [14], 2014, Indonesia	Cohort	NA/3789	At home during pregnancy (After delivery)	1.16 (0.78–1.71) Odds Ratio	Birth order, maternal age at delivery, father’s education, household income, and residence
Qiu [9], 2014, China	Cohort	1009/10095	Any place during pregnancy At home during pregnancy (After delivery)	1.12 (0.95–1.32) 1.17 (0.98–1.41) Odds Ratio	Maternal age, educational level, employment status, preeclampsia, diabetes, parity, history of preterm delivery, and cesarean delivery
Miyake [10], 2013, Japan	Cohort	138/1565	At home during pregnancy At work during pregnancy (Before delivery)	0.91 (0.48–1.65) 0.97 (0.36–2.23) Odds Ratio	Maternal age, region of residence, number of children, family structure, maternal education, maternal employment, alcohol consumption during the preceding month, BMI, and baby’s gender
Luo [11], 2012, China	Case control	198/524	Any place during pregnancy (After delivery)	2.20 (1.56–3.12) Odds Ratio	Delivery data, family income, maternal age, education level, and pre-pregnancy BMI
Khader [12], 2011, Jordan	Cross sectional	1173/8490	Any place during pregnancy (After delivery)	1.61 (1.30–1.99) Odds Ratio	Maternal age, level of education, employment, family income, height, blood type, parity, history of preterm delivery
Ashford [13], 2010, USA	Cross sectional	43/210	Any place during pregnancy (After delivery)	2.30 (0.96–5.96) Odds Ratio	Age, education, ethnicity, gestational age, and prenatal conditions
Fantuzzi [26], 2007, Italy	Case control	299/855	At home during pregnancy (After delivery)	0.92 (0.65–1.31) Odds Ratio	Maternal age, previous preterm deliveries, hypertension, diabetes, antenatal class attendance and moderate physical activity
Wu [28], ^‡^ 2007, China	Cohort	17/384	Any place during pregnancy (Before delivery)	1.13 (0.37–3.42) Odds Ratio	NA
Wu [29],^‡^ 2007, China	Cohort	80/1388	At home during pregnancy (Before delivery)	1.19 (0.75–1.87) Odds Ratio	NA
Ward [27], 2007, United Kingdom	Cohort	1171/18,297	At home during pregnancy (After delivery)	1.21 (0.96–1.51) Odds Ratio	Maternal age, BMI, parity, alcohol use, maternal education, income, ethnicity, gestational diabetes
Kim [30], 2005, Korea	Cohort	NA/2645	Any place during pregnancy (Before delivery)	0.80 (0.50–1.20) Odds Ratio	Vaginal bleeding during pregnancy, alcohol abuse, prior spontaneous abortion, prior preterm delivery, prior preeclampsia, drug abuse, and housework
Goel [31], 2004, India	Cross sectional	105/576	Any place during pregnancy (After delivery)	1.15 (0.69–1.92) Odds Ratio	Maternal age, education, occupation, birth order, number of live issues and anemia
Jaakkola [32], * 2001, Finland	Cohort	16/389	Any place during pregnancy At home during pregnancy At work during pregnancy (Before delivery)	1.95 (0.48–7.91) 0.65 (0.06–6.81) 2.35 (0.50–11.1) Odds Ratio	Sex, birth order, maternal age, BMI before pregnancy, marital status, index of socioeconomic status, alcohol consumption during pregnancy, and employment during pregnancy
Windham [37], * 2000, USA	Cohort	256/4454	Any place during pregnancy (Before delivery)	1.19 (0.86–1.63) Odds Ratio	Prior pregnancy history, race, BMI, life events and education
Pichini [41], ^‡^ 2000, Spain	Cohort	23/429	Any place during pregnancy (Before delivery)	0.91 (0.32–2.59) Odds Ratio	NA
Hanke [33], * 1999, Poland	Cross sectional	95/1751	At home during pregnancy (After delivery)	1.27 (0.84–1.94) Odds Ratio	Maternal age, mean height, parity, and infant's sex
Sadler [34],^‡^ 1999, USA	Nested cohort	56/2283	Any place during pregnancy (Before delivery)	0.67 (0.35–1.30) Odds Ratio	NA
Ahluwalia et al [35], ^†^ 1997, USA	Cohort	NA/17412	Any place during pregnancy (After delivery)	1.28 (0.64–2.58) Odds Ratio	Ethnicity, education, marital status, parity, state, alcohol use, weight gain, pre-pregnancy BMI, and altitude
Eskenazi [51], 1995, USA	Cross sectional	257/3529	Any place during pregnancy (Before delivery)	1.02 (0.51–2.03) Relative Risk	NA
Ewko [36],^‡^ 1993, USA	Case control	368/368	At home during pregnancy (After delivery)	1.50 (1.03–2.19) Odds Ratio	Age, race and parity
Mathai [42],^‡^ 1992, India	Cohort	48/994	At home during pregnancy (Before delivery)	1.56 (0.86–2.83) Odds Ratio	NA
Ahlborg et al [52], 1991, Sweden	Cohort	109/2940	Any place during pregnancy At home during pregnancy At work during pregnancy (Before delivery)	0.84 (0.53–1.33) 0.49 (0.23–1.06)1.27 (0.70–2.31) Risk Ratio	Maternal age, previous spontaneous abortion, educational level, working status, planning of pregnancy, and frequency of alcohol use, parity and place of residence
Lazzaroni [43],^‡^ 1990, Italy	Cohort	25/1004	Any place during pregnancy (After delivery)	1.08 (0.47–2.49) Odds Ratio	NA
Martin [44],^‡^ 1986, USA	Cohort	121/3891	Any place during pregnancy (Before delivery)	1.00 (0.68–1.47) Odds Ratio	NA

BMI, body mass index; CI, confidence interval; NA, not available.

* Risk estimates were converted by the method proposed by Harmling et al [38].

^†^ Risk estimates were summarized by the random-effect model [46].

^‡^ OR and 95% CI were calculated from published data with EpiCalc 2000 software (version 1.02; Brixton Health).

Characteristics related to study quality are summarized in Tables 2–4. Briefly, 6 cohort studies [9,14,27,32,35,43] were not assigned a star because preterm birth was not presented at the start of study; 3 prospective studies [28,34,37] were not assigned a star because the follow-up rate was less than 70%; 5 cohort studies [10,14,27,32,52] were assigned 2 stars because they adjusted for several important confounders in the primary analyses; and 2 case-control studies [11,36] were not assigned a star because the controls of their study did not come from the same population as the study group. None of the cross-sectional studies [12–13,31,33,51] described any assessments undertaken for ensuring quality assurance or clarifying the percentage of patients for which data was incomplete.

**Table 2:** Methodological quality of cohort studies included in the meta-analysis*.

First author (reference), publication year	Representativenessof the exposed cohort	Selection of the unexposed cohort	Ascertainment of exposure	Outcome of interest not present at start of study	Control for important factor or additional factor^†^	Assessment of outcome	Adequacy of follow-up of cohorts^‡^
Andriani, 2014	⚝	⚝	⚝	—	⚝⚝	⚝	⚝
Qiu, 2014	⚝	⚝	⚝	—	⚝	⚝	⚝
Miyake, 2013	⚝	⚝	⚝	⚝	⚝⚝	⚝	⚝
Wu, 2007	⚝	⚝	⚝	⚝	—	⚝	—
Wu, 2007	⚝	⚝	⚝	⚝	—	⚝	⚝
Ward, 2007	⚝	⚝	⚝	—	⚝⚝	⚝	⚝
Kim, 2005	⚝	⚝	⚝	⚝	—	⚝	⚝
Jaakkola, 2001	⚝	⚝	⚝	—	⚝⚝	⚝	⚝
Windham, 2000	⚝	⚝	⚝	⚝	⚝	⚝	—
Pichini, 2000	⚝	⚝	⚝	⚝	—	⚝	⚝
Sadler, 1999	⚝	⚝	⚝	⚝	—	⚝	—
Ahluwalia, 1997	⚝	⚝	⚝	—	⚝	⚝	⚝
Mathai, 1992	⚝	⚝	⚝	⚝	—	⚝	⚝
Ahlborg, 1991	⚝	⚝	⚝	⚝	⚝⚝	⚝	⚝
Lazzaroni, 1990	⚝	⚝	⚝	—	—	⚝	⚝
Martin, 1986	⚝	⚝	⚝	⚝	—	⚝	⚝

* A study could be awarded a maximum of one star for each item except for the item Control for important factor or additional factor. The definition/explanation of each column of the Newcastle-Ottawa Scale is available from (http://www.ohri.ca/programs/clinical_epidemiology/oxford.asp.).

† A maximum of 2 stars could be awarded for this item. Studies that controlled for maternal age received one star, whereas studies that controlled for other important confounders such as body mass index, parity received an additional star.

‡ A cohort study with a follow-up rate >70% was assigned one star.

**Table 3:** Methodological quality of case-control studies included in the meta-analysis*.

First author (reference), publication year	Adequate definition of cases	Representativeness of cases	Selection of control subjects	Definition of control subjects	Control for important factor or additional factor^†^	Exposure assessment	Same method of ascertainment for all subjects	Non-response Rate^‡^
Luo, 2012	⚝	⚝	—	⚝	⚝⚝	⚝	⚝	⚝
Fantuzzi, 2007	⚝	⚝	⚝	⚝	⚝	⚝	⚝	⚝
Ewko, 1993	⚝	⚝	—	⚝	⚝⚝	⚝	⚝	⚝

* A study could be awarded a maximum of one star for each item except for the item Control for important factor or additional factor. The definition/explanation of each column of the Newcastle-Ottawa Scale is available from (http://www.ohri.ca/programs/clinical_epidemiology/oxford.asp.).

† A maximum of 2 stars could be awarded for this item. Studies that controlled for maternal age received one star, whereas studies that controlled for other important confounders such as body mass index, parity received an additional star.

‡ One star was assigned if there was no significant difference in the response rate between control subjects and cases by using the chi-square test (*P*>0.05).

**Table 4:** Methodological quality of cross-sectional studies included in the meta-analysis*.

Item/Study	Khader, 2011			Ashford, 2010			Goel, 2004			Hanke, 1999			Eskenazi, 1995		
	Yes	No	Unclear	Yes	No	Unclear	Yes	No	Unclear	Yes	No	Unclear	Yes	No	Unclear
1) Define the source of information (survey, record review)	√			√			√			√			√		
2) List inclusion and exclusion criteria for exposed and unexposed subjects (cases and controls) or refer to previous publications	√			√				√		√			√		
3) Indicate time period used for identifying patients	√				√			√		√			√		
4) Indicate whether or not subjects were consecutive if not population-based	√			√			√			√			√		
5) Indicate if evaluators of subjective components of study were masked to other aspects of the status of the participants	√			√			√			√			√		
6) Describe any assessments undertaken for quality assurance purposes (e.g., test/retest of primary outcome measurements)		√			√			√			√			√	
7) Explain any patient exclusions from analysis		√		√				√			√		√		
8) Describe how confounding was assessed and/or controlled.	√			√			√			√				√	
9) If applicable, explain how missing data were handled in the analysis	√				√			√		√			√		
10) Summarize patient response rates and completeness of data collection	√				√			√		√			√		
11) Clarify what follow-up, if any, was expected and the percentage of patients for which incomplete data or follow-up was obtained		√			√			√			√			√	

* The definition/explanation of each column of the Agency for Healthcare Research and Quality is available from (http://www.ahrq.gov/research/findings).

### Passive maternal smoking exposure

**Fig 2:**
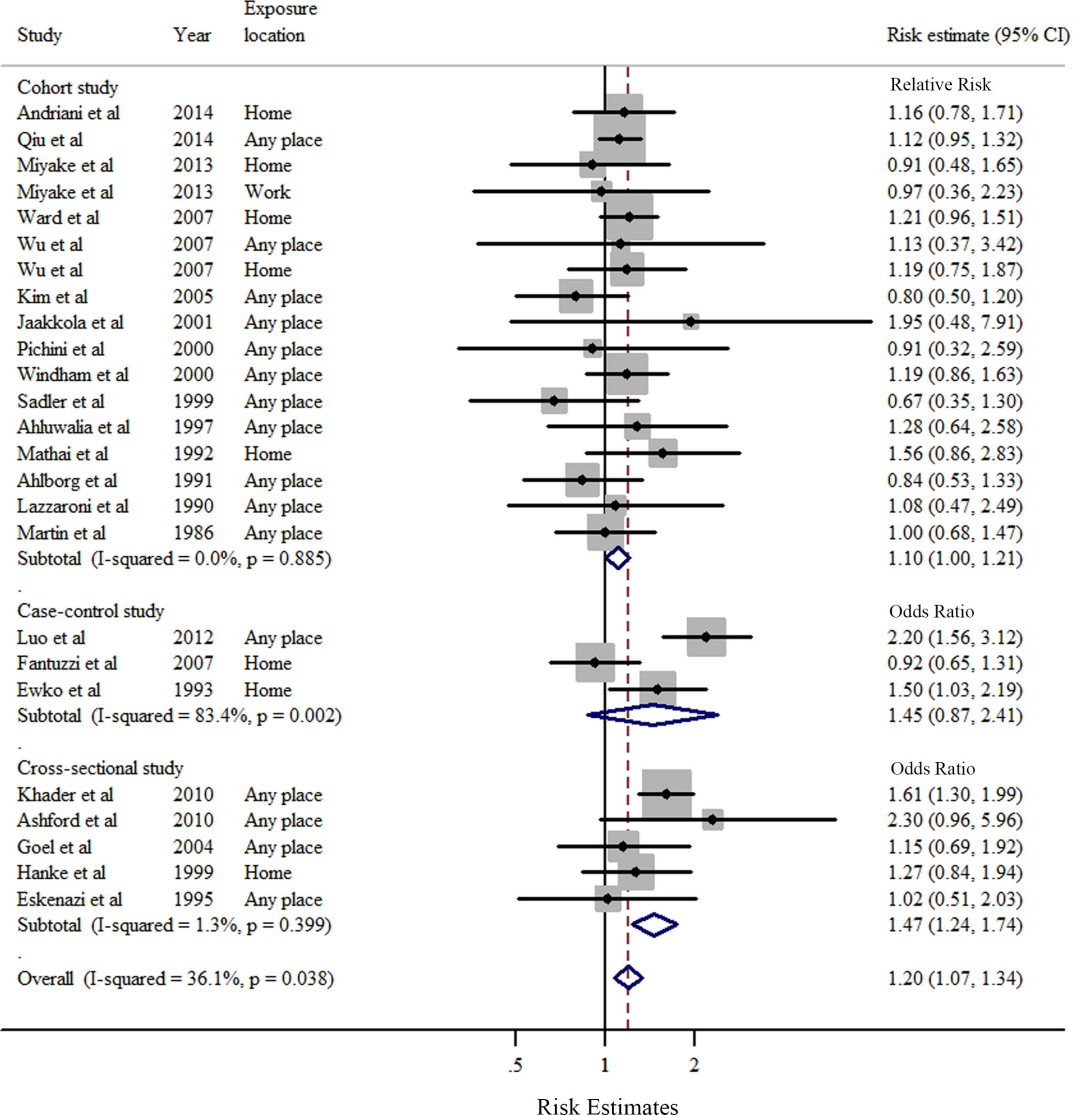
Forest plots (random effect model) of meta-analysis on the relationship between passive maternal smoking and preterm birth by study design. Squares indicate study-specific risk estimates (size of the square reflects the study-specific statistical weight); horizontal lines indicate 95% CIs; diamond indicates the summary risk estimate with its 95% CI.

**Fig 3:**
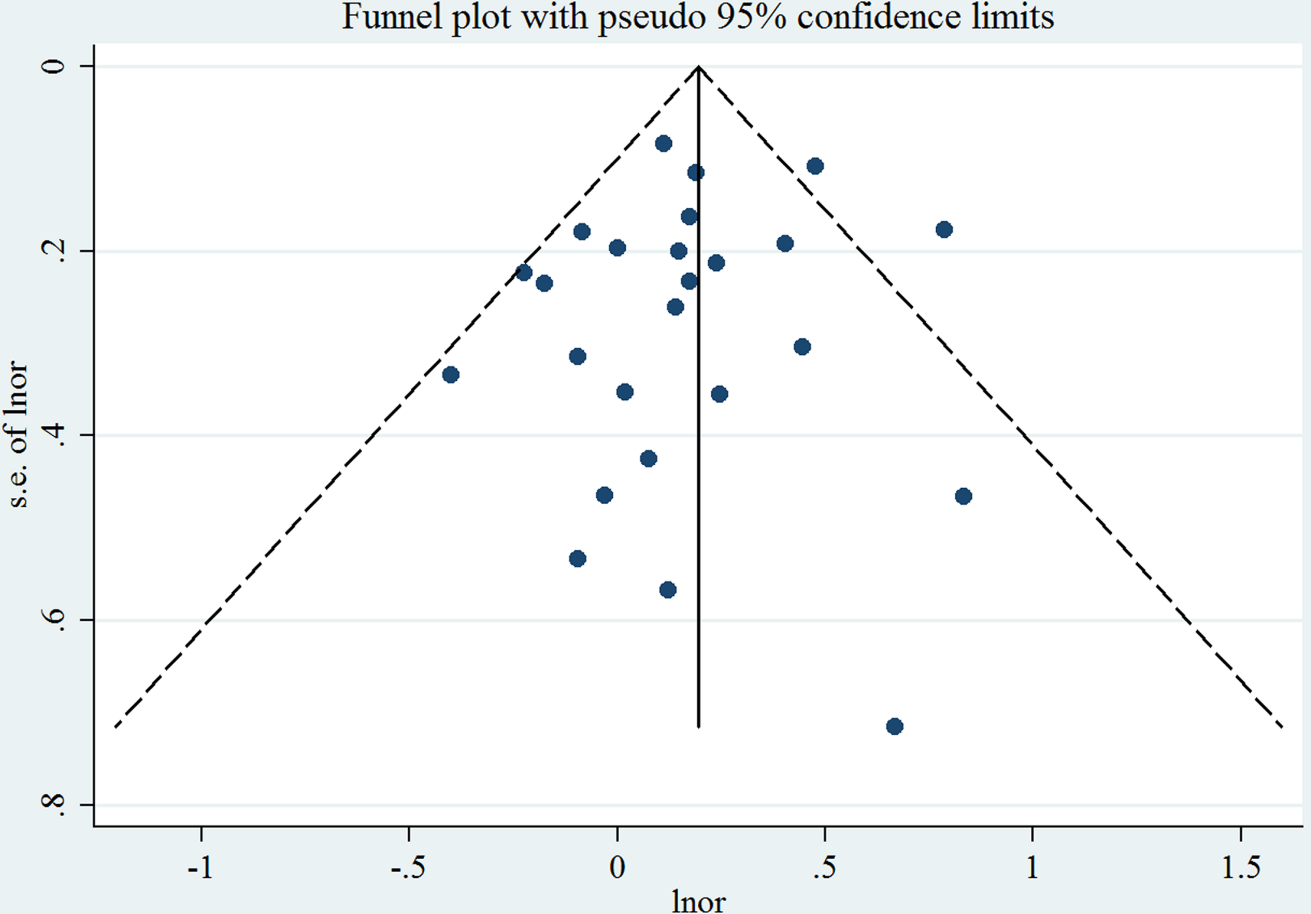
Funnel plot corresponding to the random-effects meta-analysis of the relationship between passive maternal smoking and preterm birth.

**Fig 4:**
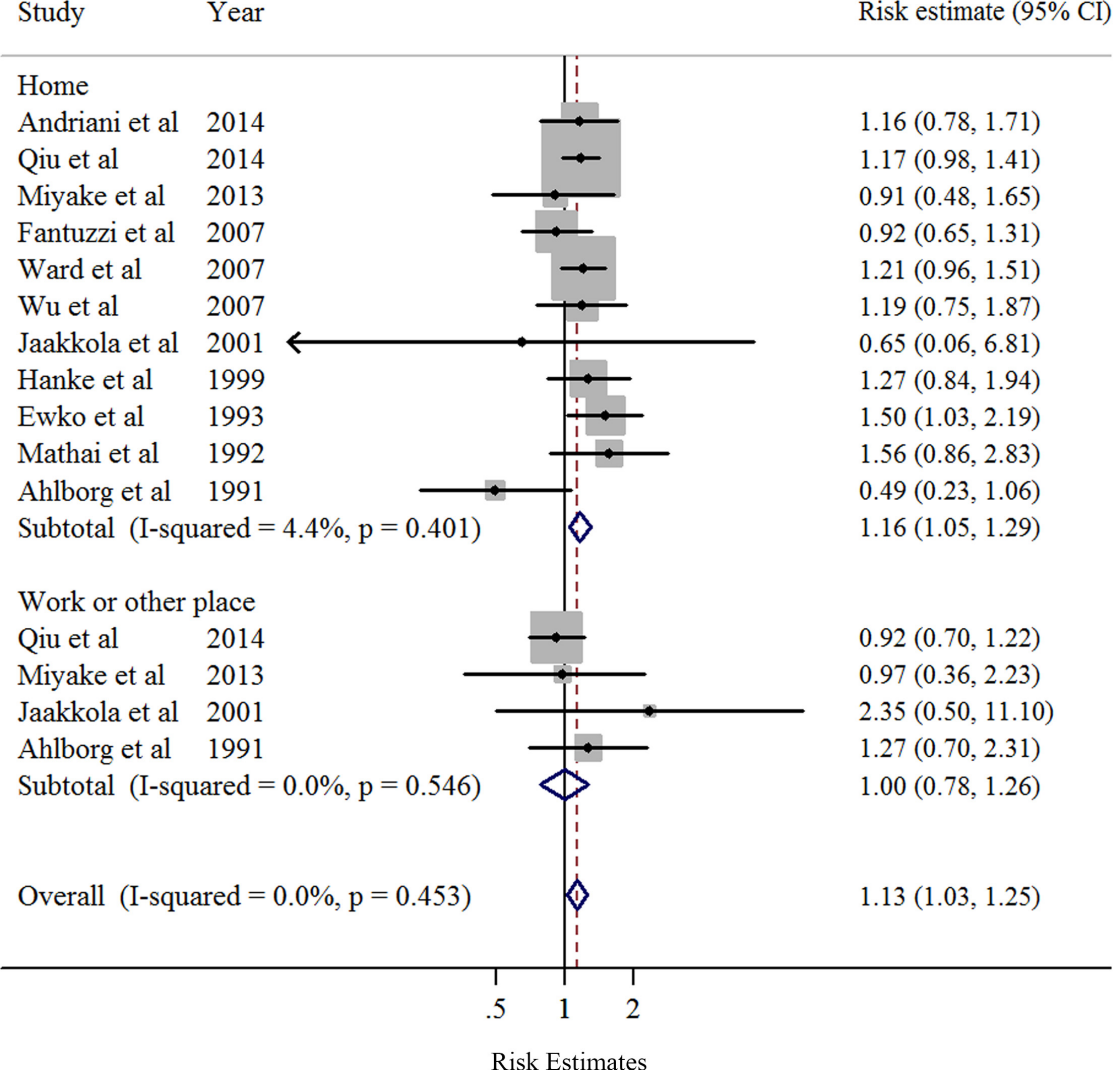
Forest plots (random effect model) of meta-analysis on the relationship between passive maternal smoking and preterm birth by exposure location. Squares indicate study-specific risk estimates (size of the square reflects the study-specific statistical weight); horizontal lines indicate 95% CIs; diamond indicates the summary risk estimate with its 95% CI.

Overall, compared to women who were never exposed to passive smoking during pregnancy, women who had ever been exposed had a significantly increased risk of preterm birth (SOR = 1.20, 95% CI: 1.07–1.34), with moderate heterogeneity (*I*^2^ = 36.1%, *P* = 0.038) (Table 5 and Fig 2). Publication bias was not observed according to Egger’s test (*P* = 0.51) or Begg's test (*P* = 0.91), and no asymmetry was noted in the funnel plot upon visual inspection (Fig 3). When the association was examined according to exposure location (Fig 4), a significantly increased risk of preterm birth was associated with exposure to passive smoking at home (SOR = 1.16, 95% CI: 1.04–1.30), with little heterogeneity (*I*^2^ = 4.4%, *P* = 0.401). We found no significant association between passive smoking at work or another place and preterm birth.

**Table 5:** Summary risk estimates of the associations between passive maternal smoking and preterm birth.

	No. of	SOR	95%CI	*Q*	*I*^2^	*P*_h_^†^
	Study			statistics	(%)	
**Overall**	24	1.20	1.07–1.34	37.6	36.1	0.038
**Exposed at home**	11	1.16	1.05–1.29	10.5	4.4	0.401
**Exposed at work or other places**	4	1.00	0.78–1.26	2.1	0	0.546
**Subgroup Analyses**						
**Study Design**						
Cohort study	16	1.10	1.00–1.21	9.6	0	0.885
Cross-sectional study	5	1.47	1.24–1.74	4.1	1.3	0.399
Case-control study	3	1.45	0.87–2.41	12.1	83.4	0.002
**Study Location**						
Asia	10	1.26	1.05–1.52	23.3	57.1	0.010
North America	7	1.18	0.99–1.41	7.4	18.9	0.285
Europe	7	1.10	0.94–1.29	4.2	0	0.647
**Number of Cases**						
≥100	12	1.22	1.05–1.41	27.2	55.9	0.007
<100	9	1.21	0.97–1.51	2.0	0.2	0.367
Unknown	3	1.02	0.78–1.34	6.6	0	0.581
**Time of Exposure Measurement**						
Before delivery (all studies)	11	1.02	0.87–1.18	7.0	0	0.803
After delivery (all studies)	13	1.33	1.15–1.53	23.1	48.1	0.027
Before delivery (cohort studies)	10	1.01	0.87–1.18	7.0	0	0.730
After delivery (cohort studies)	6	1.16	1.03–1.31	0.9	0	0.967
**Adjustment for Potential Confounders**						
**Maternal Age**						
Yes	13	1.27	1.09–1.47	27.7	53.1	0.010
No	11	1.06	0.91–1.25	6.3	0	0.789
**SES/Education**						
Yes	12	1.28	1.10–1.49	23.9	49.8	0.021
No	12	1.08	0.94–1.25	10.1	0	0.525
**Alcohol Drinking**						
Yes	6	1.07	0.90–1.26	5.2	0	0.524
No	18	1.25	1.10–1.43	29.4	42.2	0.031
**Body Mass Index**						
Yes	6	1.33	1.04–1.71	11.5	47.6	0.076
No	17	1.18	1.08–1.29	24.2	29.9	0.113
**Parity**						
Yes	10	1.28	1.15–1.43	10.7	6.9	0.378
No	13	1.16	0.96–1.40	25.0	48.0	0.023
**Preeclampsia**						
Yes	2	1.07	0.92–1.25	2.0	49.7	0.158
No	21	1.23	1.09–1.39	32.3	31.8	0.073

CI, confidence interval; SES, socioeconomic status; SOR, summarized odds ratio.

^**†**^ *P* value for heterogeneity within each subgroup.

### Subgroup and sensitivity analyses

The results of stratified analyses according to study characteristics and adjustments for potential confounders are presented in Table 5. When stratified by study design, the SORs for cohort, cross-sectional, and case-control studies were 1.10 (95% CI = 1.00–1.23, *I* ^2^ = 0%), 1.47 (95% CI = 1.23–1.74, *I*^2^ = 1.3%), and 1.45 (95% CI = 0.87–2.41, *I*^2^ = 83.4%), respectively (Fig 2). A significant positive association between passive smoking and preterm birth was observed for studies conducted in Asia, with an SOR of 1.26 (95% CI = 1.05–1.52). In the subgroup analysis stratified by the time of exposure measurement, we observed a significant association in studies that measured passive smoking exposure after delivery, but not in those that measured passive smoking before delivery. Additionally, the significant association between passive maternal smoking and increased risk of preterm birth was consistently observed in studies with more than 100 cases of preterm birth and in studies adjusted for maternal age, socioeconomic status and/or education, body mass index, and parity (Table 5).

**Fig 5:**
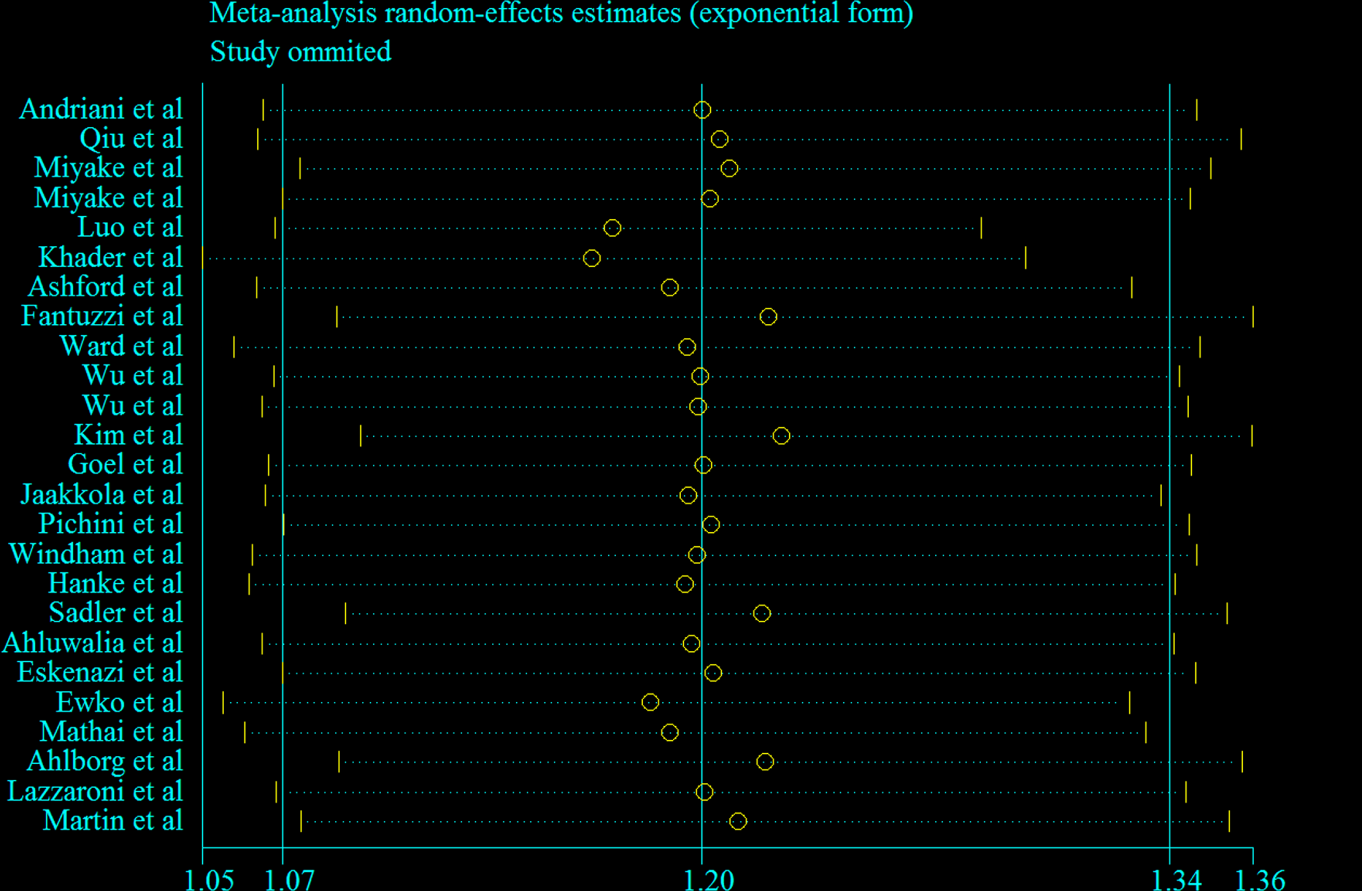
Sensitivity plot corresponding to the relationship between passive maternal smoking and preterm birth.

Fig 5 visually depicts the results of the sensitivity analysis. The SORs ranged from 1.16 (95% CI = 1.05–1.29, *I*^2^ = 22.9%) after omission of the study by Khader et al [12] to 1.24 (95% CI = 1.11–1.38, *I*^2^ = 33.9%) after omission of the study by Kim et al [30]. Additionally, we excluded 2 studies [33,37] in which risk estimates were recalculated by the effective-count method proposed by Hamling et al [38]; this result was robust (SOR = 1.21, 95% CI = 1.07–1.37, *I*^2^ = 42.6%). Lastly, we excluded 8 studies [28–29,34,36,41–44] that provided crude risk estimates without adjustment for any potential confounders; this result was also robust (SOR = 1.23, 95% CI = 1.07–1.42, *I*^2^ = 50.9%).

## Discussion

The rate of preterm birth has increased in most countries in the past decade and it represents an important public health issue. A previous meta-analysis of studies published prior to 2009 found no significant association between passive maternal smoking and risk of preterm birth [8]. For this report, we conducted an updated meta-analysis, which indicates that exposure to passive maternal smoking at any place and at home increases the risk of preterm birth by 20% and 16%, respectively. These findings were robust among cohort and cross-sectional studies. Additionally, significant associations between passive maternal smoking and preterm birth were observed in studies conducted in Asia and in studies adjusted for maternal age, socioeconomic status and/or education, body mass index, and parity (Table 5).

When passive smoking and preterm birth were examined according to exposure location, a statistically significant association was found only for passive smoking exposure at home (Table 5). Since only 4 studies [9–10,32,52] that provided risk estimates of exposure to passive maternal smoking at work or another place were included in this analysis, the results of the current meta-analysis partly support the hypothesis that, compared with exposure to smoke from people at work or another place, there is a greater risk of preterm birth associated with exposure to smoke from family members at home. However, only 2 of the included studies evaluated the dose-dependent association between passive smoking and preterm birth in their primary analyses [9,14]. Specifically, Andriani et al [14] found that, in both urban and rural areas, the risk estimates for preterm birth of infants born after paternal smoking exposure were stronger with the increasing number of cigarettes consumed by the father [9].

In the subgroup analyses stratified by study design, we found that the point estimate of the relationship between preterm birth and passive smoking was slightly stronger among cross-sectional studies and weaker among cohort studies. Compared with cross-sectional or retrospective studies, prospective studies had fewer biases due to their prospective nature. However, we observed non-significant associations between passive smoking and preterm birth among studies that collected exposure information before delivery. This same pattern was observed when we restricted the stratified analysis to cohort studies (Table 5). This issue might be attributed to the difference between study design and the time of data collection of several included studies. For example, Qiu et al [9] conducted a birth cohort study from 2010 to 2012. Nevertheless, trained study interviewers conducted in-person interviews to the majority of women (84%) within 1 to 3 days after delivery using a standardized and structured questionnaire. Similar procedures were used in several included cohort studies [14,27,32,35,43]; although the authors conducted prospective studies of newborns, information regarding passive maternal smoking was collected after the preterm birth regardless of the period between data collection and outcome. By comparison, Miyaka et al [10] reported the relationship between passive maternal smoking and preterm birth on the basis of a prospective pre-birth cohort study in Japan. The data of passive maternal smoking was collected with the first questionnaire, which was completed before delivery. Thus, recall bias could be largely ruled out in this study. Future prospective cohort studies that collect exposure data at the first prenatal visit are warranted to confirm these findings.

A significant association between passive maternal smoking at any place and preterm birth risk was only observed in studies from Asia (Table 5), which could be attributed to higher rates of passive smoking exposure and preterm birth in these populations. The mean preterm birth rates in cohort and cross-sectional studies of the included studies were 9.1%, 8.3%, and 4.8% for Asia, North America, and Europe, respectively. The mean passive maternal smoking exposure rates in epidemiologic studies were 44%, 26.4%, and 37.4% for Asia, North America, and Europe, respectively. The significant associations we observed could be the result of the larger sample sizes of the studies in Asia (n = 2768) compared with the studies in North America (n = 1101) and Europe (n = 1738).

Several potential biological mechanisms have been suggested to explain the positive association between passive maternal smoking and preterm birth. Passive smoke contains several toxic chemicals, including nicotine, carbon monoxide, and DNA adducts [53–55]. Previous experimental studies suggested that nicotine and carbon monoxide in the blood not only decrease blood flow between the uterus and the placenta but also influence the development of the fetus and the placenta [53–55]. Carbon monoxide is a potent vasoconstrictor of placental vessels and it can integrate with oxygen to form carboxyhemoglobin, which may restrict the amount of oxygen supplied to the fetus and cause low fetal tissue oxygenation [14]. Moreover, Jauniaux et al [56] demonstrated that the toxic chemicals in passive smoke could regulate protein metabolism and enzyme activity through interfere with the trophoblastic and biological functions of fetal cells, which may lead to restricted fetal growth and preterm birth. These mechanisms may be the foundation of the association between passive maternal smoking and risk of preterm birth.

Our current meta-analysis has several strengths. First, to the best of our knowledge, this is the most comprehensive and current meta-analysis for evaluating the association between passive maternal smoking and preterm birth. Second, our meta-analysis included 24 observational studies, 16 of which were cohort studies, that involved 5607 patients from a total population of approximately 88,200 participants; this provided sufficient power to detect modest associations. Third, compared with previous meta-analyses [8,57], we conducted more subgroup and sensitivity analyses to explore the heterogeneity among results and to validate the findings of this study.

Several potential limitations of this meta-analysis should be acknowledged. First, considering the nature of observational studies, we could not fully rule out the possibility of residual confounding. When we assessed the quality of the included studies, only 5 cohort studies [10,14,27,32,52] adjusted for more than 2 important potential confounders. The results were robust after excluding these studies [28–29,34,36,41–44], which provided crude risk estimates without adjustment for any potential confounders, but these studies accounted for one-third of all included studies. We did not have access to the primary data for these studies. Future prospective cohort studies are necessary to fully adjust for the potential confounders and report analyses stratified by possible risk factors to rule out residual confounding. Second, self-reported passive maternal smoking during pregnancy was not validated by objective measurements such as serum cotinine levels or nicotine levels in the hair, which might result in misclassification. For example, DeLorenze et al [58] suggested that self-administered questionnaires could underestimate low levels of passive maternal smoking. Additionally, after prospectively investigating 94 mother-infant pairs, Eliopoulos et al [59] provided evidence that cotinine concentrations in newborn hair might be a validated biomarker for determining the intensity of passive maternal smoking. However, Pickett et al [60] suggested that there was a high correlation between urinary cotinine measurements and the self-reported number of cigarettes to which pregnant women in the United States were exposed at any given time point, which suggests that it is reasonable to use self-reported data. Only 2 studies included in our meta-analysis provided both self-reported and biochemically validated exposure data (serum cotinine or nicotine in hair) in the primary analysis, but the results were inconsistent between passive maternal smoking and preterm birth [11,32]. For example, when the serum cotinine cut-off level was set at 3 ng/ml, relatively high concordance between self-reported passive smoking and serum cotinine was reported in the study of Luo et al (Kappa-value = 0.75) [11]. Additionally, the results of Jaakkola et al [32] showed that the concentrations of nicotine in the hair of women whose spouse was a current smoker were substantially higher than in women who reported no exposure either at home or at work (medians concentrations: 1.32 vs. 0.61 μg/g). Therefore, future prospective cohort studies should use both self-reported and validated biomarkers to confirm our findings. Third, several included studies performed stratified analyses according to the subtype of preterm birth (medically indicated or spontaneous) [9,30], the time of preterm birth (extremely, very, or moderate) [9,37], the period of passive smoking exposure during pregnancy (first, second, or third trimester) [9–10,32], and the dose-response analysis of passive maternal smoking. However, since few studies provided this information, we did not perform subgroup analyses according to these variables.

In conclusion, in this updated and comprehensive meta-analysis, we found that women who had ever been exposed to passive maternal smoking at any place or at home had a significantly higher risk of preterm birth than women who had never been exposed to passive smoking. Future prospective cohort studies are warranted to examine potential confounders of this association and to provide more detailed results that are stratified by passive maternal smoking in different trimesters and by different types and causes of preterm birth.

## Supporting Information

S1 Checklist The PRISMA checklist for this meta-analysis. (DOC)

S1 Database The database of the analysis between passive maternal smoking during pregnancy and preterm birth. (XLSX)
